# Plasma HERV-K envelopE RNA: a minimally invasive biomarker for lung adenocarcinoma detection and prognostic assessment in the context of conventional serum tumor markers

**DOI:** 10.3389/fimmu.2026.1782414

**Published:** 2026-07-01

**Authors:** Fan Bu, Zi-Yan Jin, Zhi-Qiang Ling

**Affiliations:** 1 Zhejiang Cancer Institute, Zhejiang Cancer Hospital, Hangzhou, Zhejiang, China; 2 The Second Clinical Medical College of Zhejiang Chinese Medicine University, Hangzhou, Zhejiang, China

**Keywords:** biomarker, diagnosis, HERV-K env mRNA, liquid biopsy, lung adenocarcinoma (LUAD), prognosis

## Abstract

**Introduction:**

Early detection of lung adenocarcinoma (LUAD) remains challenging because low-dose computed tomography produces many false-positive pulmonary nodules and conventional serum tumor markers show histology-dependent diagnostic performance. We investigated whether plasma HERV-K env mRNA could serve as a minimally invasive biomarker for subtype-specific diagnosis and prognostic assessment in lung cancer.

**Methods:**

This retrospective single-center study enrolled 379 subjects, including 249 patients with non-small cell lung cancer (162 LUAD and 87 lung squamous cell carcinoma [LUSC]), 30 patients with malignant pulmonary nodules, 50 patients with benign pulmonary nodules, and 50 healthy controls. HERV-K env mRNA was quantified in tumor tissues, paired adjacent tissues, and EDTA-anticoagulated plasma by qRT-PCR. Associations with clinicopathological variables, tissue-plasma concordance, correlations with conventional serum tumor markers, ROC diagnostic performance, Kaplan-Meier survival, and multivariable Cox regression were analyzed.

**Results:**

HERV-K env mRNA was significantly upregulated in NSCLC tissues versus paired adjacent tissues (P < 0.0001) and was higher in LUAD than in LUSC (P < 0.0001). Tissue expression increased with advanced T stage, lymph node metastasis, and higher TNM stage (all P < 0.0001). Plasma HERV-K env mRNA levels were significantly elevated in lung cancer compared with benign pulmonary nodules and healthy controls (P < 0.0001) and correlated strongly with tissue expression in both LUSC (r = 0.9025) and LUAD (r = 0.7305). ROC analysis showed excellent performance for stage I/II LUAD versus healthy controls (AUC = 0.914), moderate performance for malignant pulmonary nodules versus healthy controls (AUC = 0.758), but limited performance for early-stage LUSC (AUC = 0.505). Plasma HERV-K env mRNA showed no significant correlations with conventional serum tumor markers. High plasma HERV-K env mRNA was associated with poorer overall survival in LUAD (P = 0.035), and multivariable Cox regression supported independent prognostic value in the overall cohort.

**Discussion:**

Plasma HERV-K env mRNA captures a biologically plausible and analytically non-redundant signal with the strongest current clinical promise in LUAD rather than LUSC. Its LUAD performance appears competitive with published single-marker serum assays, but broader clinical application and comparison with optimized conventional multi-marker panels require prospective multicenter and same-cohort validation.

## Introduction

Lung adenocarcinoma (LUAD) has become the most prevalent histological subtype of lung cancer, accounting for over 40% of all cases. In recent years, its incidence has continued to rise globally, posing a significant clinical challenge (1). Early detection of lung adenocarcinoma is key to improving patient prognosis, significantly increasing the 5-year survival rate for Stage I patients to 68%~92% (2). However, current diagnostic strategies for lung cancer are hampered by critical drawbacks. As reported in the literature, low-dose computed tomography screening is plagued by a false-positive rate exceeding 95% for indeterminate pulmonary nodules (3). Meanwhile, established serum biomarkers such as CEA, CYFRA21-1, SCC-Ag, NSE, and CA125 show moderate and histology-dependent diagnostic performance in published NSCLC cohorts. In a large suspected-lung-cancer cohort, single-marker AUCs for NSCLC versus benign lung disease were 0.86 for CYFRA21-1, 0.80 for CEA, and 0.85 for NSE, whereas a three-marker combination reached 0.92 (4). In a separate NSCLC subtype cohort including benign chest disease comparators, single-marker AUCs in LUAD were 0.85 for CEA, 0.79 for CYFRA21-1, and 0.69 for CA125, whereas in LSCC they were 0.93 for CYFRA21-1, 0.78 for SCC-Ag, 0.70 for CEA, and 0.68 for CA125 (5).

Liquid biopsy technology, centered on circulating RNA, has emerged as a promising research direction to address the current diagnostic challenges associated with blood tumor biomarkers (6–9). Despite its promising prospects, there is currently a lack of clinically validated, reliable biomarkers, which constitutes a critical bottleneck in translating this technology into routine clinical practice (10). The reactivation of Human Endogenous Retrovirus K is a process driven by epigenetic dysregulation during cellular carcinogenesis, which leads to the production of its envelope proteins (11). These proteins have been demonstrated to possess significant oncogenic properties (12). Although studies have reported the role of HERV-K envelope protein in other malignancies such as melanoma and breast cancer, its potential as a biomarker for diagnosing LUAD via liquid biopsy technology still awaits further rigorous validation (13).

We hypothesize that plasma HERV-K envelope mRNA may serve as a novel and sensitive biomarker for lung adenocarcinoma-focused detection. To test this hypothesis, our study was designed to quantify and compare its expression levels in matched lung cancer tissue and plasma samples, to examine subtype-specific diagnostic performance in LUAD and LUSC, and to compare its analytical independence from conventional serum biomarkers in lung cancer.

## Materials and methods

### Patients and samples

**Figure 1:**
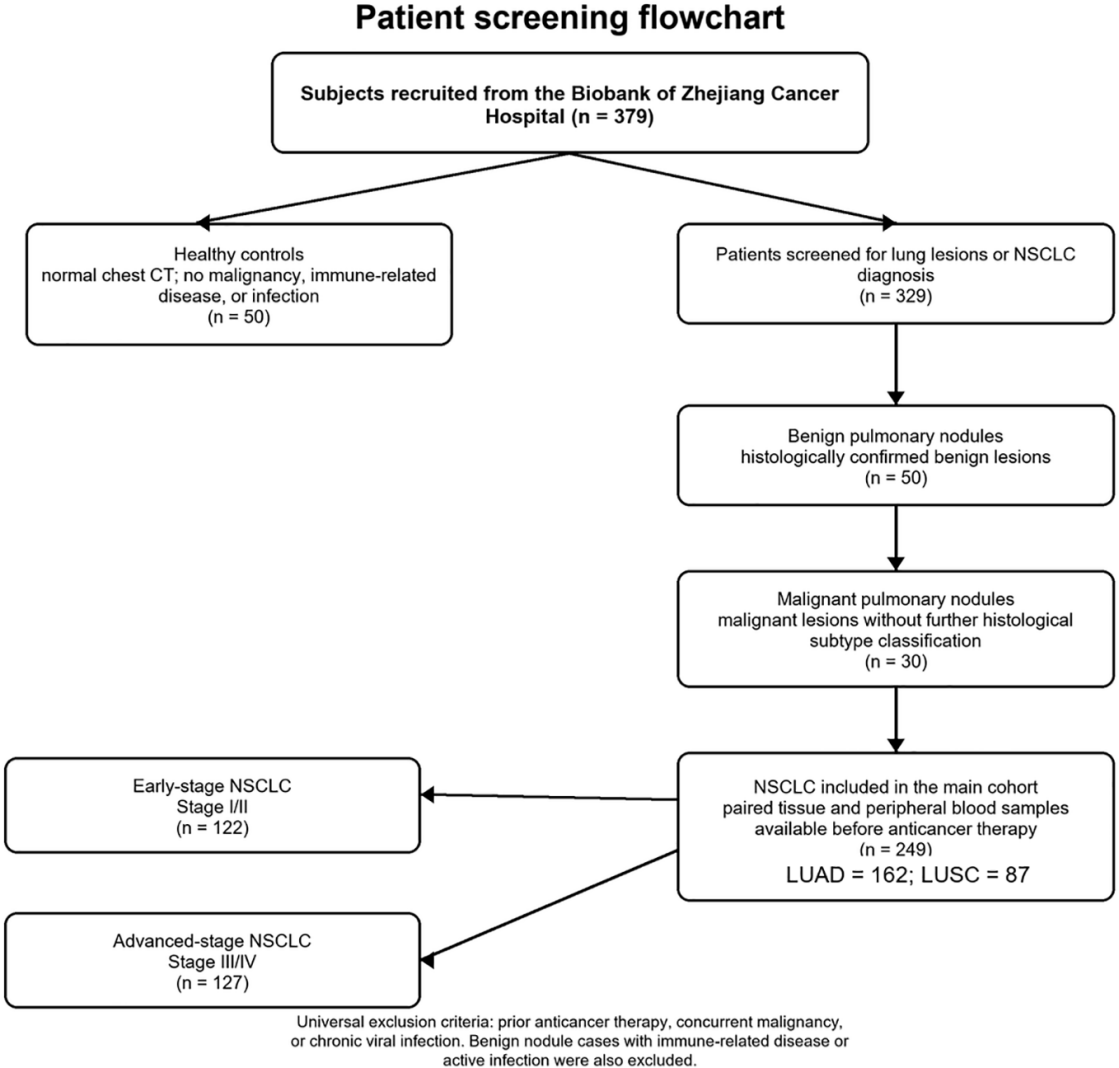
Patient screening flowchart of the retrospective study cohort. A total of 379 subjects were screened from the Biobank of Zhejiang Cancer Hospital, including healthy controls, benign pulmonary nodules, malignant pulmonary nodules, and the NSCLC cohort used for tissue and plasma analyses. Malignant pulmonary nodules represent malignant lesions confirmed pathologically but not further subclassified into LUAD or LUSC in the present dataset. Universal exclusion criteria included prior anticancer therapy, concurrent malignancy, or chronic viral infection.

A total of 379 subjects were retrospectively recruited for this study from the Biobank of Zhejiang Cancer Hospital (patient screening summarized in **Figure 1**). The study cohort included 249 patients with non-small cell lung cancer (NSCLC), 30 patients with histologically diagnosed malignant pulmonary nodules, 50 patients with histologically confirmed benign pulmonary nodules, and 50 age- and sex-matched healthy volunteers. For all participants, universal exclusion criteria included prior anticancer therapy, concurrent malignancy, or chronic viral infection. In addition, patients with benign pulmonary nodules were excluded if they had immune-related diseases or active infection. Healthy controls were required to have normal chest CT findings and no history of malignancy, immune-related disease, or infection. Among the 249 patients with NSCLC, there were 162 cases of lung adenocarcinoma (LUAD) and 87 cases of lung squamous cell carcinoma (LUSC). Based on disease stage, 122 cases were categorized as treatment-naive early-stage disease (stages I/II) and 127 cases as advanced-stage disease (stages III/IV). Malignant pulmonary nodules were defined as pathologically confirmed malignant lesions that were not further subclassified into LUAD or LUSC in the present plasma diagnostic comparison dataset.

Tumor tissues, paired adjacent normal tissues, and peripheral blood samples were collected from patients with NSCLC, whereas only peripheral blood samples were obtained from patients with benign pulmonary nodules, malignant pulmonary nodules, and healthy controls. Peripheral blood samples were processed to obtain both serum and EDTA-anticoagulated plasma. Serum samples were used to measure conventional clinical tumor markers according to standard clinical laboratory protocols, whereas EDTA-anticoagulated plasma was used for HERV-K envelope RNA quantification to preserve RNA integrity. This design allowed paired evaluation of tissue-plasma concordance in the NSCLC cohort while separately examining the ability of plasma HERV-K env mRNA to distinguish malignant pulmonary nodules from benign nodules and healthy controls.

Commercial Human Universal cDNA (Takara, Japan) was served as the reference tissue control. It is typically reverse transcribed from pooled RNA of over 30 human tissues to encompass multi-organ transcriptomes. The reference blood control was prepared from pooled peripheral blood of 10 age- and sex-matched blood donors.

### RNA preparation and cDNA synthesis

Total RNA was extracted from lung cancer tumor tissues, paired adjacent non-cancerous tissues, and EDTA-anticoagulated plasma using TRIzol reagent (Invitrogen) according to the manufacturer’s protocol. RNA purity and concentration were verified using a NanoDrop 2000 spectrophotometer (Thermo Fisher Scientific), with an acceptable A260/A280 ratio range of 1.8-2.1 for qualified samples. RNA integrity was further assessed using an Agilent 2100 Bioanalyzer, requiring an RNA Integrity Number (RIN) ≥ 7 for all samples. Genomic DNA contamination was eliminated by DNase I treatment (RNase-free DNase Set, Qiagen). First-strand cDNA synthesis was performed using PrimeScript RT Master Mix (Takara Bio) with random hexamer and oligo-dT primers, using 1 µg of total RNA in a 20 µL reaction volume. The thermal cycling conditions were as follows: 37 °C for 15 minutes for genomic DNA removal, 42 °C for 50 minutes for reverse transcription, and 70 °C for 15 minutes for enzyme inactivation. Negative controls without reverse transcriptase (-RT) were included for each sample set. The synthesized cDNA was stored at -80 °C until analysis.

### Real-time quantitative polymerase chain reaction

Real-time quantitative PCR (qRT-PCR) was performed using SYBR Green chemistry to assess the expression abundance of HERV-K env in the samples. The primer sequences used were as follows: forward 5’-CAC AAC TAA AGA AGC TGA CG-3’ and reverse 5’-CAT AGG CCC AGT TGG TAT AG-3’ (GenBank Accession No. AC074261 from Chr12q14.1), while *GAPDH* served as the endogenous control (forward: 5’-CTC TCT GCT CCT CCT GTT CG-3’; reverse: 5’-ACG ACC AAA TCC GTT GAC TC-3’; GenBank accession No.NM_002046.5). Reactions contained 10 μL 2 × SYBR Premix Ex Taq (Takara Bio), 0.8 μL each primer (10 μM), 2 μL cDNA template, and nuclease-free water to 20 μL final volume. Amplification was performed on a QuantStudio 5 system (Applied Biosystems) under the following standardized conditions: initial denaturation at 95 °C for 30 seconds, followed by 40 cycles of 95 °C for 5 seconds and 60 °C for 30 seconds (with fluorescence acquisition), and concluding with a melt curve analysis (from 65 °C to 95 °C at an increment of 0.5 °C per second). All samples were run in technical triplicates alongside no-template controls (NTCs) and no-reverse-transcription controls (NRTs). Expression quantification was calculated using the 2^⁻ΔΔCt^ method after confirming amplification efficiency (90–105%) and single-peak melt curves, which verified amplicon specificity.

### Statistical methods

Statistical analyses were performed using SPSS 21.0 (SPSS Inc., IL, USA) and GraphPad Prism 5 (La Jolla, CA, USA). For normally distributed data, group comparisons were conducted using one-way ANOVA and independent-sample t-tests. For non-normally distributed data, the Mann-Whitney U test and Kruskal-Wallis H test were applied. Spearman’s rank correlation was used for correlation analysis, and associations between categorical variables were assessed using the chi-square test or Fisher’s exact test. ROC curves were used to summarize diagnostic discrimination with AUC values. To address the reviewer-requested comparison with conventional serum tumor markers, we additionally performed an indirect literature-contextual comparison using PubMed-verified NSCLC studies that reported ROC/AUC metrics for CEA, CYFRA21-1, NSE, SCC-Ag, and CA125. Because the available raw workbook did not retain the exact original ROC modeling objects and did not include conventional serum-marker measurements for benign-nodule or healthy-control comparators, cutoff values, ROC P-values, and direct within-cohort marker-versus-marker AUC comparisons could not be re-derived with sufficient certainty from the raw spreadsheets alone. Accordingly, the revised manuscript presents validated AUC-based interpretation for HERV-K env mRNA together with an explicitly indirect literature-based ROC comparison for conventional markers. Statistical significance was defined as P < 0.05.

## Results

### Analysis of HERV-K env RNA expression in NSCLC tissues

**Figure 2:**
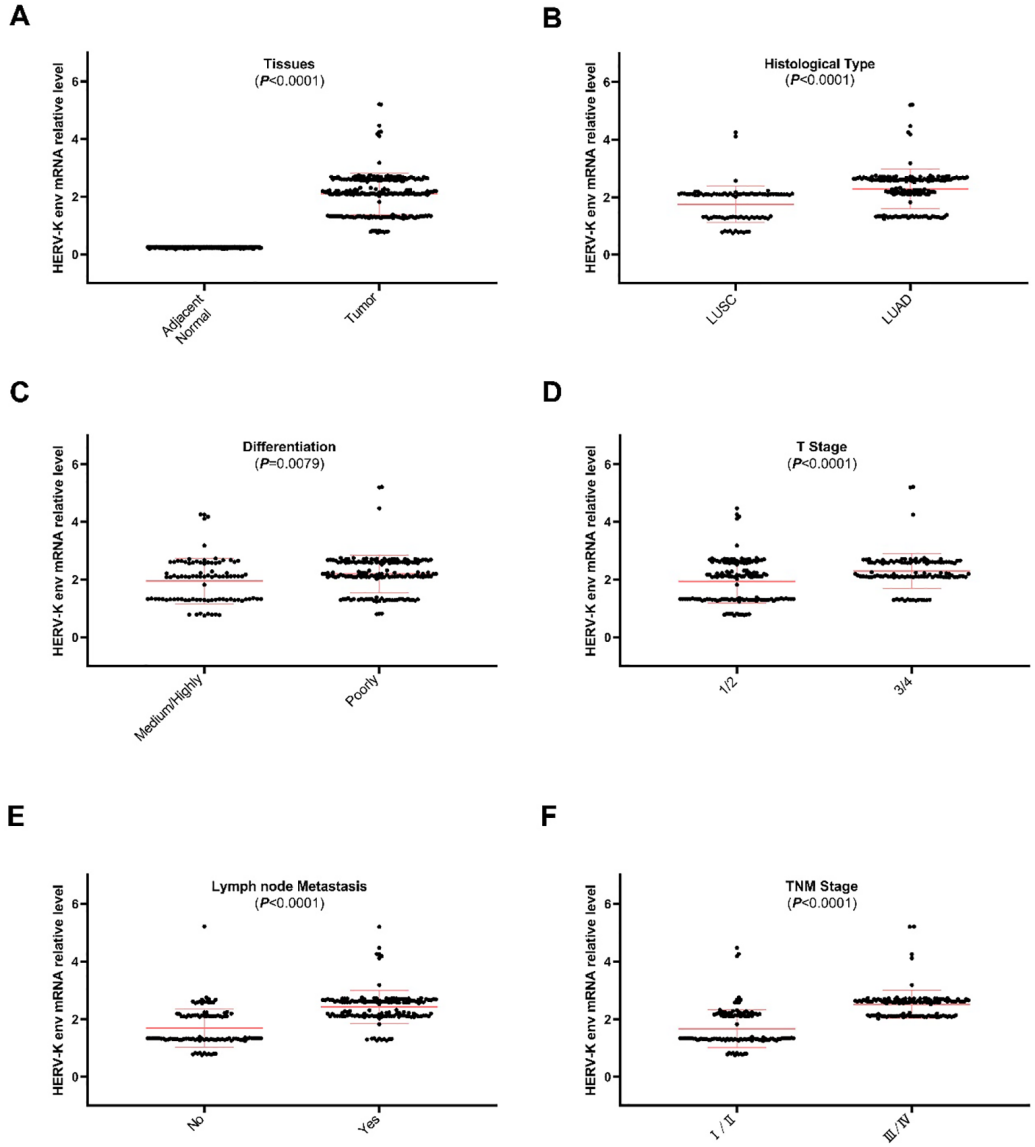
Analysis of HERV-K env mRNA expression levels in lung cancer tissues. **(A)** Comparison of HERV-K env mRNA expression levels between tumor tissues and matched adjacent normal tissues. **(B)** Comparison of HERV-K env mRNA expression levels across histological subtypes of lung cancer. **(C)** Comparison of HERV-K env mRNA expression levels according to tumor differentiation grade. **(D)** Comparison of HERV-K env mRNA expression levels stratified by T classification. **(E)** Comparison of HERV-K env mRNA expression levels in relation to lymph node metastasis status. **(F)** Comparison of HERV-K env mRNA expression levels according to TNM stage classification. Data are presented as mean ± SEM. Statistical significance was determined using unpaired Student’s t-test for two-group comparisons, with *P* < 0.05 considered statistically significant.

We first assessed the expression levels of HERV-K env mRNA in non-small cell lung cancer (NSCLC) tissues and their matched adjacent normal tissues. As shown in **Figure 2A**, the levels of HERV-K env mRNA were significantly higher in NSCLC tissues compared to the paired adjacent normal tissues (*P* < 0.0001), suggesting its potential involvement in the pathogenesis and progression of NSCLC. Furthermore, HERV-K env mRNA was significantly upregulated across all pathological stages (I-IV), indicating its association with lung carcinogenesis and disease progression. Notably, the expression level of HERV-K env mRNA was significantly higher in lung adenocarcinoma (LUAD) than in lung squamous cell carcinoma (LUSC) (*P* < 0.0001, **Figure 2B**), implying a more prominent role in LUAD. In the NSCLC cohort, poorly differentiated tumors exhibited significantly higher levels of HERV-K env mRNA than moderately/well-differentiated tumors (*P* = 0.0079, **Figure 2C**). Moreover, the expression of HERV-K env mRNA increased significantly with disease progression: it was higher in T3/T4-stage tumors than in T1/T2-stage tumors (*P* < 0.0001, **Figure 2D**), higher in node-positive tumors than in node-negative tumors (*P* < 0.0001, **Figure 2E**), and higher in advanced-stage tumors (III/IV) than in early-stage tumors (I/II) (*P* < 0.0001, **Figure 2F**), suggesting a potential role in tumor progression and metastasis.

Analysis of clinical data from 249 patients revealed no significant associations between tissue HERV-K env mRNA expression and gender (P = 0.176), age at diagnosis (P = 0.631), smoking history (P = 0.897), tumor size (P = 0.228), tumor location (P = 0.804), or EGFR mutation status (P = 0.495) (**Table 1**). When lymph node status was further reviewed by specific N stage, the distribution remained descriptively skewed toward high HERV-K env expression across N0, N1, and N2 groups, but the overall N-stage comparison was not statistically significant in the available cohort-level contingency analysis (**Table 1**).

**Table 1:** Correlation between tissue HERV-K env level and clinical characteristics in NSCLC.

Clinical characteristics	Expression		χ^2^	*P*
	High	Low/normal		
Gender				
Male	139	26	1.835	0.176
Female	76	8		
Age at diagnosis				
<70	67	12	0.231	0.631
≥70	148	22		
Smoking status				
Non-smoker	86	14	0.017	0.897
Smoker	129	20		
Histological type				
LUAD	153	9	25.796	<0.0001
LUSC	62	25		
Tumor size				
<5	142	26	1.453	0.228
≥5	73	8		
Differentiation				
Medium/High	75	17	4.779	0.029
Poorly	142	14		
Tumor location				
Left	90	15	0.061	0.804
Right	125	19		
T stage				
1/2	113	25	9.118	0.003
3/4	105	6		
N stage				
N0	94	12	1.348	0.510
N1	58	9		
N2	62	13		
TNM stage				
I/II	91	31	36.859	<0.0001
III/IV	127	0		
EGFR mutation status				
Wild-type	196	41	0.466	0.495
Mutant-type	9	3		

Pearson’s chi-square test. High expression in test tissue samples was defined as at least 1.3-fold upregulation relative to Commercial Human Universal cDNA, determined using the median method. For the present revision, lymph node status is additionally summarized by N stage (N0, N1, and N2), and the overall contingency comparison was not statistically significant (P = 0.510).

### Analysis of HERV-K env RNA expression in lung cancer plasma and its correlation with patient clinicopathological parameters

**Figure 3:**
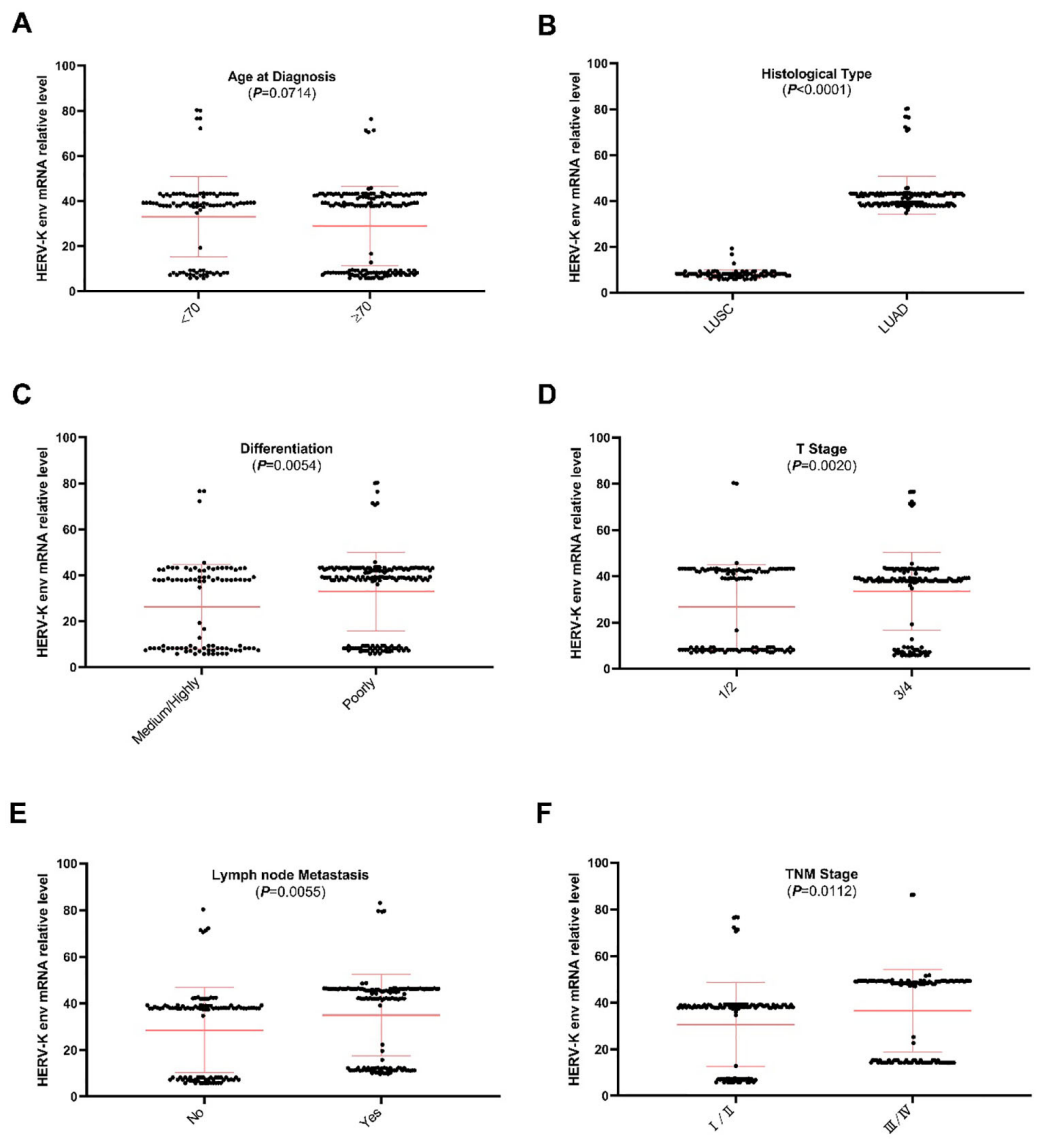
Analysis of the correlation between plasma HERV-K env mRNA expression levels and clinicopathological features in lung cancer patients. **(A)** Association between age at diagnosis and plasma HERV-K env mRNA levels. **(B)** Comparison of plasma HERV-K env mRNA expression levels across different histological subtypes. **(C)** Comparison of plasma HERV-K env mRNA expression levels stratified by tumor differentiation grade. **(D)** Comparison of plasma HERV-K env mRNA expression levels according to T stage classification. **(E)** Association between lymph node metastasis status and plasma HERV-K env mRNA levels. **(F)** Comparison of plasma HERV-K env mRNA expression levels across TNM stages. Data are presented as mean ± SEM. Statistical analyses were performed using unpaired Student’s t-test for comparisons between two groups. A *P*-value less than 0.05 was considered statistically significant.

Plasma HERV-K env mRNA levels were significantly elevated in patients with lung cancer (including both LUAD and LUSC subtypes) compared with individuals with benign pulmonary nodules and healthy controls (P < 0.0001). Further analysis of clinicopathological correlations is summarized in **Table 2**. In the cohort of 249 patients, no significant associations were observed between plasma HERV-K env mRNA levels and gender (P = 0.305), tumor location (P = 0.724), tumor size (P = 0.630), smoking history (P = 0.425), or EGFR mutation status (P = 0.813). Significant associations were identified with histological subtype (P = 0.004), tumor differentiation (P = 0.003), T stage (P = 0.002), binary lymph node metastasis status (P < 0.0001), and TNM stage (P = 0.005). When nodal involvement was reviewed using specific N stages, high plasma HERV-K env mRNA expression was observed across N0, N1, and N2 groups, although the overall N-stage comparison was not statistically significant in the currently available cohort-level contingency analysis (**Figure 3** and **Table 2**).

**Table 2:** Correlation between plasma HERV-K env level and clinical characteristics in NSCLC.

Clinical characteristics	Expression		χ^2^	*P*
	High	Low/normal		
Gender				
Male	111	54	1.053	0.305
Female	51	33		
Age at diagnosis				
<70	58	21	3.555	0.059
≥70	104	66		
Smoking status				
Non-smoker	68	32	0.635	0.425
Smoker	94	55		
Histological type				
LUAD	134	28	8.260	0.004
LUSC	58	29		
Tumor size				
<5	111	57	0.232	0.630
≥5	51	30		
Differentiation				
Medium/High	49	43	8.729	0.003
Poorly	112	44		
Tumor location				
Left	67	38	0.125	0.724
Right	95	49		
T stage				
1/2	78	60	9.928	0.002
3/4	84	27		
N stage				
N0	66	40	1.825	0.402
N1	48	19		
N2	47	28		
TNM stage				
I/II	74	48	7.940	0.005
III/IV	98	29		
EGFR mutation status				
Wild-type	150	87	0.056	0.813
Mutant-type	8	4		

Pearson’s chi-square test. High expression was defined as at least 34.71-fold upregulation in test plasma samples relative to reference blood controls, calculated using the median method. For the present revision, lymph node status is additionally summarized by N stage (N0, N1, and N2), and the overall contingency comparison was not statistically significant (P = 0.402).

### Plasma HERV-K env mRNA as an independent biomarker for lung cancer: correlation with tissue expression and conventional serum markers

**Figure 4:**
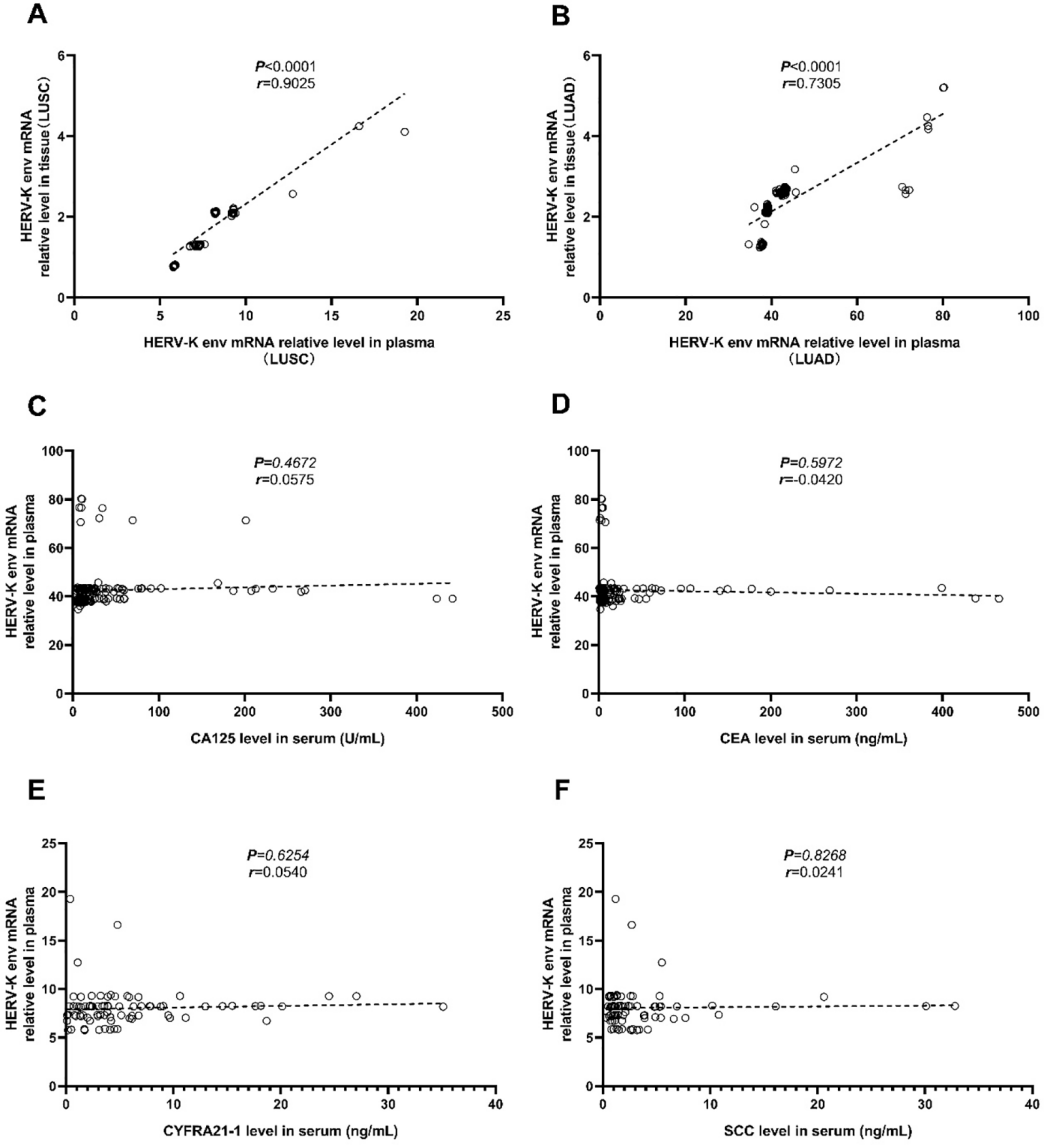
Analysis of the correlation between plasma HERV-K env mRNA expression levels, tumor tissue expression levels, and conventional serum tumor markers in lung cancer patients. **(A)** Correlation between plasma and tumor tissue HERV-K env mRNA levels in patients with lung squamous cell carcinoma (LUSC). **(B)** Correlation between plasma and tumor tissue HERV-K env mRNA levels in patients with lung adenocarcinoma (LUAD). **(C)** Correlation between serum CA125 levels and plasma HERV-K env mRNA levels in LUAD patients. **(D)** Correlation between serum CEA levels and plasma HERV-K env mRNA levels in LUAD patients. **(E)** Correlation between serum CYFRA21–1 levels and plasma HERV-K env mRNA levels in LUAD patients. **(F)** Correlation between serum SCC levels and plasma HERV-K env mRNA levels in LUSC patients. Correlations were analyzed using Pearson’s correlation coefficient **(r)**, with two-tailed P-values indicating statistical significance. Linear regression was performed to visualize relationships with dashed regression lines.

We evaluated the potential of plasma HERV-K env mRNA as an independent biomarker by analyzing its correlation with tumor tissue expression and conventional serum tumor markers in lung cancer patients. The study showed significant positive correlations between plasma HERV-K env mRNA expression levels and tissue HERV-K env mRNA expression levels in both LUSC (*r* = 0.9025, *P* < 0.0001, **Figure 4A**) and LUAD (*r* = 0.7305, *P* < 0.0001, **Figure 4B**), supporting its diagnostic potential.

Further analysis revealed no significant correlations between plasma HERV-K env mRNA abundance and existing serum tumor markers. In LUAD patients, no significant associations were found with CA125 (r = 0.0575, P = 0.4672, **Figure 4C**), CEA (r = -0.0420, P = 0.5972, **Figure 4D**), or CYFRA21-1 (r = 0.0540, P = 0.6254, **Figure 4E**). Similarly, in LUSC patients, no significant correlation was observed with SCC antigen levels (r = 0.0241, P = 0.8268, **Figure 4F**). These findings indicate analytical non-redundancy rather than superiority over conventional serum tumor markers. The available raw workbook contained serum-marker measurements for the NSCLC cohort but not corresponding benign-nodule or healthy-control marker datasets, so verified head-to-head ROC comparisons could not be completed in this revision. To address the reviewers’ concern as rigorously as possible, we therefore contextualized the HERV-K env mRNA ROC results against PubMed-verified published NSCLC cohorts reporting AUCs for CEA, CYFRA21-1, NSE, SCC-Ag, and CA125.

### Plasma HERV-K env mRNA as a potential biomarker for diagnosis and prognosis in lung cancer: a lung cancer subtype-specific analysis

**Figure 5:**
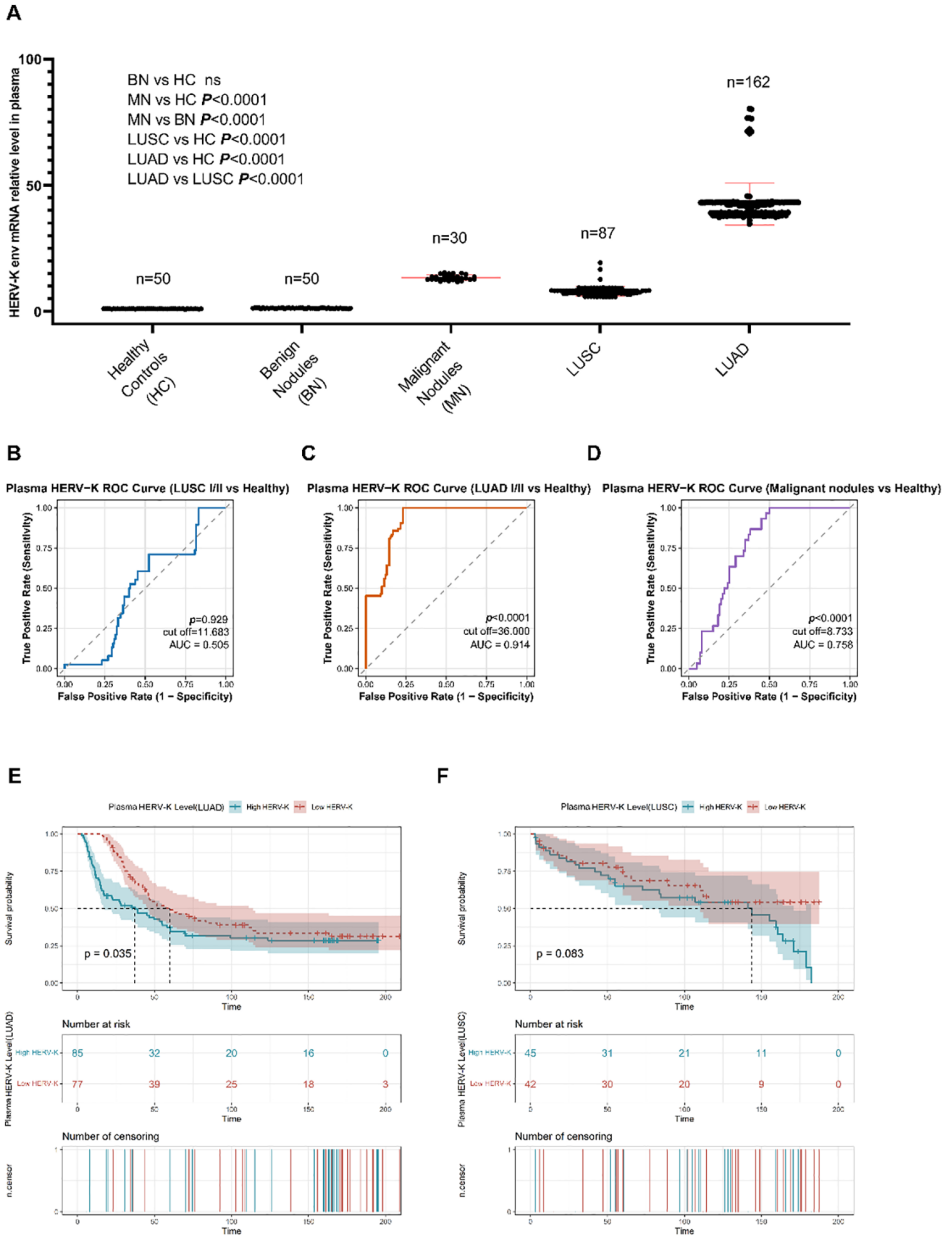
Performance of plasma HERV-K env mRNA in diagnosis and prognosis assessment with subtype-specific interpretation. **(A)** Plasma HERV-K env mRNA expression levels in healthy controls (HC), patients with benign pulmonary nodules (BN), malignant pulmonary nodules (MN), lung squamous cell carcinoma (LUSC), and lung adenocarcinoma (LUAD). In this study, malignant pulmonary nodules represent pathologically confirmed malignant lesions that were not further subclassified into LUAD or LUSC in the diagnostic comparison dataset. **(B)** ROC curve analysis of plasma HERV-K env mRNA for diagnosing early-stage squamous cell carcinoma. **(C)** ROC curve analysis of plasma HERV-K env mRNA for diagnosing LUAD. **(D)** ROC curve analysis of plasma HERV-K env mRNA for distinguishing malignant pulmonary nodules. **(E)** Kaplan-Meier survival curves comparing overall survival between LUAD patients with high and low plasma HERV-K env mRNA levels. **(F)** Kaplan-Meier survival curves comparing overall survival between LUSC patients with high and low plasma HERV-K env mRNA levels. Diagnostic performance was summarized using AUC values, and survival differences were assessed with the Kaplan-Meier method and log-rank test.

Plasma HERV-K env mRNA levels were significantly elevated in lung cancer patients (including both LUAD and LUSC subtypes) compared with individuals with benign pulmonary nodules and healthy controls (P < 0.0001, **Figure 5A**), supporting a potential role in discriminating malignant from benign pulmonary conditions. Receiver operating characteristic (ROC) curve analysis showed that plasma HERV-K exhibited differential diagnostic performance across clinical settings. Plasma HERV-K demonstrated high diagnostic accuracy for distinguishing stage I/II LUAD from healthy controls (AUC = 0.914, P < 0.0001, cut off = 36.000, **Figure 5C**) and moderate diagnostic value for differentiating malignant nodules from healthy controls (AUC = 0.758, P < 0.0001, cut off = 8.733, **Figure 5D**). In contrast, its discriminatory ability for stage I/II LUSC versus healthy controls was limited (AUC = 0.505, P = 0.929, cut off = 11.683, **Figure 5B**). At the respective cutoff values, the sensitivity and specificity were 86.8% and 21.0%; 100.0% and 76.9%; 100.0% and 50.0%. These findings suggest that plasma HERV-K may serve as a more promising biomarker for early LUAD than for early LUSC, while also providing auxiliary value in the identification of malignant pulmonary nodules. In the present dataset, the LUAD cohort included 41 Stage I cases, 11 Stage IA cases, and 19 cases with a recorded maximum tumor diameter of 2 cm or less; no LUAD cases had a recorded maximum diameter below 1 cm. These descriptive subgroup counts support the clinical relevance of earlier-stage validation, although reliable subgroup ROC recalculation was not possible from the available raw workbook alone, and radiologic ground-glass versus solid nodule annotations were not available. Published comparator studies provide useful indirect context. In a large suspected-lung-cancer cohort, CYFRA21-1, CEA, and NSE showed AUCs of 0.86, 0.80, and 0.85 for NSCLC versus benign lung disease, and the corresponding three-marker panel reached AUC 0.92 (4). In a subtype-oriented NSCLC cohort with benign chest disease comparators, LUAD was best characterized by CEA (AUC 0.85), CYFRA21-1 (AUC 0.79), and CA125 (AUC 0.69), whereas LSCC was best characterized by CYFRA21-1 (AUC 0.93), SCC-Ag (AUC 0.78), CEA (AUC 0.70), and CA125 (AUC 0.68) (5). Against this literature context, the present HERV-K env mRNA result for LUAD appears competitive with published single-marker serum assays, whereas its early-stage LUSC performance is clearly inferior to the published marker performance usually reported for LSCC. Prognostically, elevated plasma HERV-K env mRNA levels were significantly associated with reduced overall survival in LUAD (P = 0.035, **Figure 5E**), whereas the corresponding trend in LUSC did not reach statistical significance (P = 0.083, **Figure 5F**).

**Figure 6:**
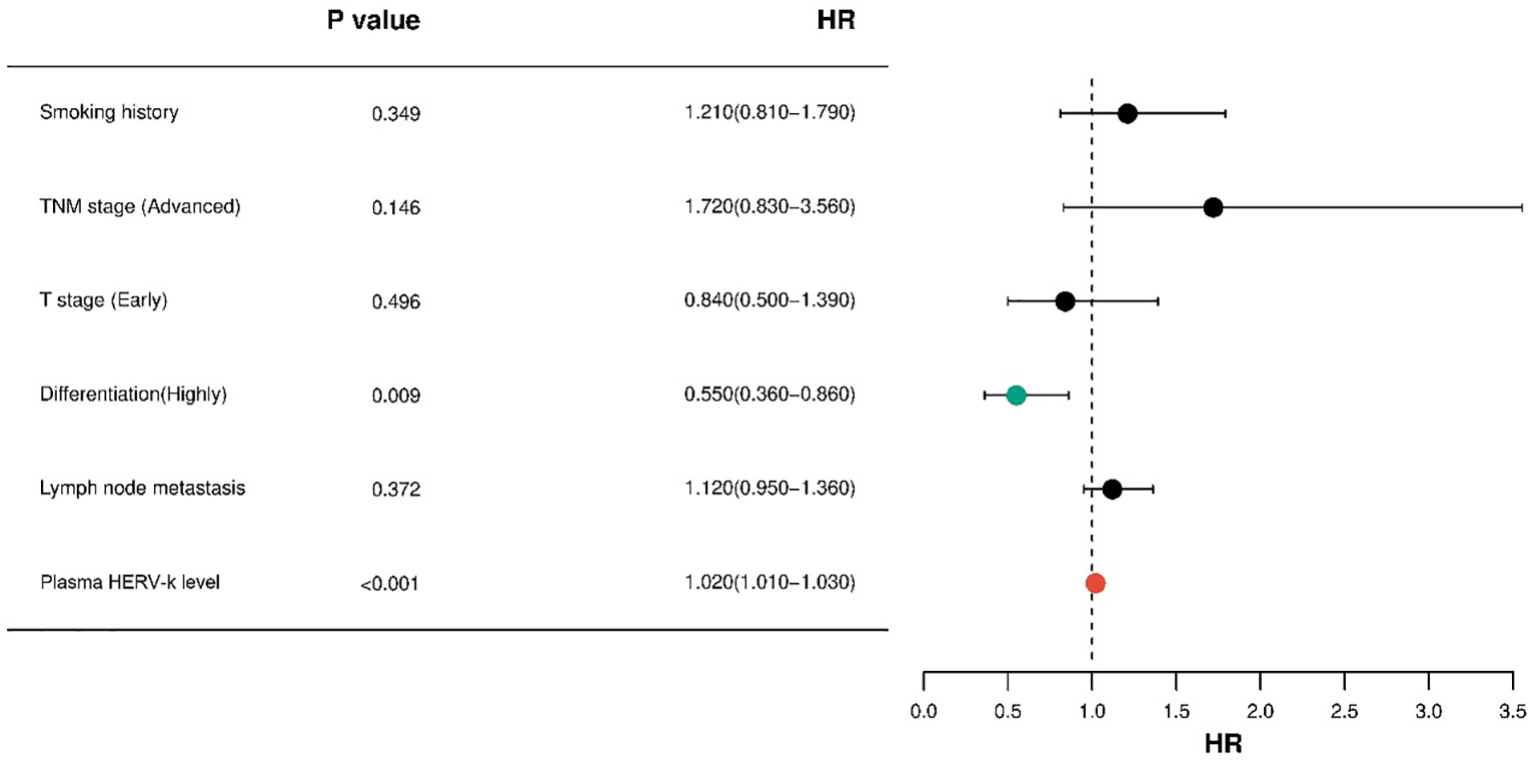
Multivariate Cox regression analysis of prognostic factors in lung cancer patients. The forest plot displays hazard ratios (HRs) and 95% confidence intervals (CIs) for variables included in the multivariate Cox proportional hazards model. The analyzed factors include TNM stage, T stage, tumor differentiation grade, lymph node metastasis status, and plasma HERV-K env mRNA expression levels. Because diagnostic performance differed substantially by histological subtype and the LUSC survival analysis was only borderline significant, the overall prognostic interpretation should be considered exploratory pending external validation.

Multivariate Cox regression analysis further supported plasma HERV-K env mRNA as an independent prognostic factor in the overall cohort (**Figure 6**). The forest plot displays hazard ratios (HRs) and 95% confidence intervals (CIs) for the evaluated variables, including TNM stage, T stage, tumor differentiation, lymph node metastasis, and plasma HERV-K env mRNA levels. In this model, an HR > 1 indicates an increased risk of mortality. Given the subtype-specific diagnostic findings and the borderline survival result in LUSC, the prognostic interpretation is presented cautiously and should be prospectively validated in independent cohorts.

## Discussion

This study demonstrates that HERV-K env mRNA is significantly upregulated in non-small cell lung cancer (NSCLC) tissues, with particularly strong expression in lung adenocarcinoma (LUAD), and that plasma HERV-K env mRNA captures a diagnostically informative signal that is most convincing in LUAD rather than uniformly across all lung cancer subtypes. The current data therefore support LUAD-focused clinical interpretation, while the evidence for early-stage LUSC detection remains limited (17–19).

Our findings align with prior evidence showing HERV-K reactivation in epithelial malignancies and extend that literature by highlighting subtype-preferential expression in LUAD relative to LUSC (20–23). The strong tissue-plasma correlations observed in both subtypes support the biological plausibility of plasma HERV-K env mRNA as a liquid biopsy analyte. However, the substantial difference between LUAD diagnostic performance (AUC = 0.914) and early-stage LUSC diagnostic performance (AUC = 0.505) indicates that any clinical application should currently be framed as LUAD-oriented rather than as a universal early lung cancer marker. The reviewer-requested comparison with conventional serum markers also becomes more nuanced after literature contextualization. Published NSCLC cohorts report single-marker AUCs in roughly the 0.80-0.86 range for CEA, CYFRA21-1, and NSE against benign comparators, whereas subtype-oriented studies report LUAD AUCs of 0.85 for CEA, 0.79 for CYFRA21-1, and 0.69 for CA125 and LSCC AUCs of 0.93 for CYFRA21–1 and 0.78 for SCC-Ag. (4, 5) In addition, paired serum-marker panels such as CYFRA21–1 plus CEA for LADC and CYFRA21–1 plus SCCAg for LSCC have been reported with AUCs of 0.891 and 0.912, respectively, underscoring the strength of optimized conventional combinations (14, 15). Within that context, the HERV-K env mRNA LUAD AUC is encouraging relative to published single-marker serum assays (16–20), but it should not be interpreted as superior to optimized conventional marker panels (21–23), especially because our comparison is cross-study rather than within-cohort. Conversely, the early-stage LUSC AUC of 0.505 falls well below the performance expected of established LSCC-oriented serum markers and further supports restricting current clinical interpretation to LUAD. Likewise, the absence of significant correlation with conventional serum tumor markers suggests non-overlapping information content rather than direct superiority. Mechanistically, HERV-K env may be involved in tumor progression through pathways such as ERK/MAPK signaling or immune modulation (24, 25), but these possibilities remain hypothetical in the absence of direct functional experiments in the present study (26, 27).

This study has several important limitations. First, the retrospective single-center design and moderate cohort size limit generalizability and create a clear need for external validation. Second, the available raw workbook did not retain the exact original ROC modeling objects or conventional-marker measurements for benign-nodule and healthy-control comparators, so ROC cutoffs, ROC P-values, and direct AUC comparisons against conventional serum tumor markers could not be re-derived with sufficient confidence from the raw spreadsheets alone; accordingly, the conventional-marker comparison presented in this revision is literature-based and indirect rather than same-cohort and head-to-head. Third, although the current cohort contained clinically relevant LUAD subsets, including Stage I, Stage IA, and 19 tumors measuring 2 cm or less, no LUAD cases with a recorded maximum diameter below 1 cm were present, and radiologic subgrouping of ground-glass versus solid nodules was not available in the raw workbook. Fourth, the mechanistic implications of HERV-K env mRNA remain speculative because no functional validation experiments were performed (28–33). Future work should therefore prioritize prospective multicenter validation, independent external cohorts, standardized assay workflows, rigorous same-cohort comparison with conventional serum biomarkers, and mechanistic studies focused on LUAD biology (34–37).

## Conclusion

HERV-K env mRNA is a promising histology-biased biomarker with the strongest current evidence in lung adenocarcinoma (LUAD). Its significant overexpression in LUAD, strong tissue-plasma concordance, and favorable LUAD diagnostic performance support further development for LUAD-focused detection and prognostic assessment. When interpreted against published ROC data for conventional NSCLC serum markers, the LUAD AUC observed here appears competitive with single-marker assays but not definitive against optimized multi-marker panels. By contrast, the present data do not support a comparable role in early-stage LUSC detection, and broader clinical translation will require prospective multicenter validation, external cohorts, and rigorous same-cohort comparison with conventional serum biomarkers.

## Data Availability

The raw data supporting the conclusions of this article will be made available by the authors, without undue reservation.
